# Putative molecular markers of *Plasmodium falciparum* resistance to antimalarial drugs in malaria parasites from Ghana

**DOI:** 10.3389/fepid.2024.1279835

**Published:** 2024-02-15

**Authors:** Sena Adzoa Matrevi, Tryphena Adams, Kwesi Zandoh Tandoh, Philip Opoku-Agyeman, Selassie Bruku, Nana Aba Ennuson, Paa Kwesi Apau-Danso, Emmanuel Fiagbedzi, Mary Avornyo, Charles James Myers, Joy Futagbi, Oheneba Charles Hagan, Benjamin Abuaku, Kwadwo Ansah Koram, Gordon Awandare, Neils Ben Quashie, Nancy Odurowah Duah-Quashie

**Affiliations:** ^1^ West African Centre for Cell Biology of Infectious Pathogens, Department of Biochemistry, Cell and Molecular Biology, University of Ghana, Accra, Ghana; ^2^ Department of Epidemiology, Noguchi Memorial Institute for Medical Research, University of Ghana, Accra, Ghana; ^3^ Department of Biochemistry, Cell and Molecular Biology, University of Ghana, Accra, Ghana; ^4^ Centre for Tropical Clinical Pharmacology and Therapeutics, University of Ghana Medical School, University of Ghana, Accra, Ghana

**Keywords:** malaria, artemisinin-based combination therapy, sulphadoxine-pyrimethamine, antimalarial drug resistance, molecular markers

## Abstract

**Introduction:**

Antimalarial drugs including artemisinin-based combination therapy (ACT) regimens and sulphadoxine-pyrimethamine (SP) are used in Ghana for malaria therapeutics and prophylaxis respectively. The genetic basis of *Plasmodium falciparum* development of drug resistance involves single nucleotide polymorphisms in genes encoding proteins for multiple cellular and metabolic processes. The prevalence of single nucleotide polymorphisms in nine *P. falciparum* genes linked to ACT and SP resistance in the malaria parasite population was determined.

**Methods:**

Archived filter paper blood blot samples from patients aged 9 years and below with uncomplicated malaria reporting at 10 sentinel sites located in three ecological zones for the Malaria Therapeutic Efficacy Studies were used. The samples used were collected from 2007-2018 malaria transmission seasons and mutations in the genes were detected using PCR and Sanger sequencing.

**Results:**

In all 1,142 samples were used for the study. For falcipain-2 gene (*pffp2*)*,* Sanger sequencing was successful for 872 samples and were further analysed. The prevalence of the mutants was 45% (392/872) with *pffp2* markers V51I and S59F occurring in 15.0% (128/872) and 3.0% (26/872) of the samples respectively. Prevalence of other *P. falciparum* gene mutations: coronin (*pfcoronin*) was 44.8% (37/90); cysteine desulfurase (*pfnfs*) was 73.9% (68/92); apicoplast ribosomal protein S10 (*pfarps10*) was 36.8% (35/95); ferredoxin (*pffd*) was 8.8% (8/91); multidrug resistance protein-1 (*pfmrp1*) was 95.2.0% (80/84); multidrug resistance protein-2 (*pfmrp2*) was 91.4% (32/35); dihydrofolate reductase (*pfdhfr*) was 99.0% (84/85); dihydropteroate synthase (*pfdhps*) was 72% (68/95).

**Discussion:**

The observation of numerous mutations in these genes of interest in the Ghanaian isolates, some of which have been implicated in delayed parasite clearance is of great interest. The presence of these genotypes may account for the decline in the efficacies of ACT regimens being used to treat uncomplicated malaria in the country. The need for continuous monitoring of these genetic markers to give first-hand information on parasite susceptibility to antimalarial drugs to inform policy makers and stakeholders in malaria elimination in the country is further discussed.

## Introduction

Malaria continues to be a major public health problem globally with high rates of morbidity and mortality in disease-endemic areas of the world. In 2022, 249 million cases and 608,000 estimated deaths occurred worldwide (1). The World Health Organization (WHO) African Malaria Region accounted for 94% and 95% respectively of the morbidity and mortality estimates stated above (1). Chemotherapy remains the mainstay of malaria control and elimination with artemisinin-based combination therapy (ACT) in disease-endemic areas (2). The reported occurrence of *Plasmodium falciparum* resistance to artemisinin (ART) in Southeast Asia (SEA) and a few countries in Africa has led to active surveillance through Therapeutic Efficacy Studies (TES) investigating clinical, *in vitro*, molecular, and pharmacokinetic analyses of antimalarial drug resistance (3).

Molecular analysis of the *P. falciparum* genome revealed mutations in the kelch propeller gene (*pfk13*) to be associated with the development of parasite tolerance to ART and are being monitored in parasite populations as markers of drug resistance (2, 4). ART resistance *pfk13* candidate mutations and novel ones have been reported in Ghanaian isolates (5–7). A study conducted in Cambodia has also shown parasites with increased ring stage survival but lacking the *pfk13* mutations which implies that there could be other genes involved and/or different resistance mechanisms (8). Some studies have suggested the contribution of multiple cellular and metabolic processes in ART resistance including hemoglobin degradation, proteotoxic/unfolded protein stress response, vesicular biogenesis as well as oxidative stress response, and mitochondrial functions (9). These studies have implicated single nucleotide polymorphisms (SNPs) in multiple genes in antimalarial drug resistance development. The antimalarial drug resistance markers are effective tools for assessing the drug resistance status of parasites for malaria control measures (3, 10, 11).

Studies have linked parasite genetic loci with *in vivo* and *in vitro* studies to ART resistance and these include missense SNPs that lead to loss of function in genetic alleles of actin-binding protein coronin (*pfcoronin* G50E, V62M, R100K, E107V, N112N/Y/D) and falcipain 2 (*pffp2* N4H, S35*, V51I, S59F, S69*, K255R) (12–15). In addition, the co-inheritance of several mutations including *P. falciparum* apicoplast ribosomal protein S10 (*pfarps10* V127M), ferredoxin (*pffd* D193Y), chloroquine resistance transporter (*pfcrt* I356T) and the multidrug resistance gene 2 (*pfmdr2* T484I) were shown to constitute a parasite genetic background (PGB) that allows for the emergence of mutations in the *pfk13* (16). Mutations in other genes, cysteine desulfurase (*pfnfs* S62N, K65Q, E67G*)* and multidrug resistance protein 1 and 2 (*pfmrp1* I876V and K1466R; *pfmrp2* N622D, I1775V, and c2397t) have also been implicated in ACT resistance (17–19). Sulphadoxine-pyrimethamine (SP) is still used for intermittent preventive treatment in pregnant women (IPTp) and seasonal malaria chemoprevention (SMC) in children in Ghana. Mutations in *P. falciparum* dihydrofolate reductase (*pfdhfr* N51I, C59R, S108N, and I164l) and dihydropteroate synthase (*pfdhps* S436A, A437G, and K540E) are implicated in SP resistance (20). More recently a mutation, *pfdhps* I431V was observed exclusively in parasites from pregnant women from Nigeria (21) and Cameroon (22, 23), however, its role in resistance is not known.

In Ghana, ACT regimens used in the treatment of uncomplicated malaria are artemether-lumefantrine (AL), artesunate-amodiaquine (AS-AQ), artesunate-pyronaridine (AS-PD), and dihydroartemisinin-piperaquine (DHAP) whilst SP is used IPTp and SMC. AS-AQ was introduced in 2005, and AL and DHAP were added in 2008 for the treatment of uncomplicated malaria. Currently, the regimens for first-line treatment are AL, AS-AQ, and AS-PD whilst DHAP is the second-line drug (24). This study assessed the prevalence of mutations in nine resistance markers (*pffp2, pfcoronin, pfnfs, pfarps10, pffd, pfmrp1, pfmrp2, pfdhfr, and pfdhps*) in the *P. falciparum* population in Ghana as part of ongoing antimalaria drug efficacy/resistance surveillance studies.

## Materials and methods

### Study sites and samples

**Figure 1:**
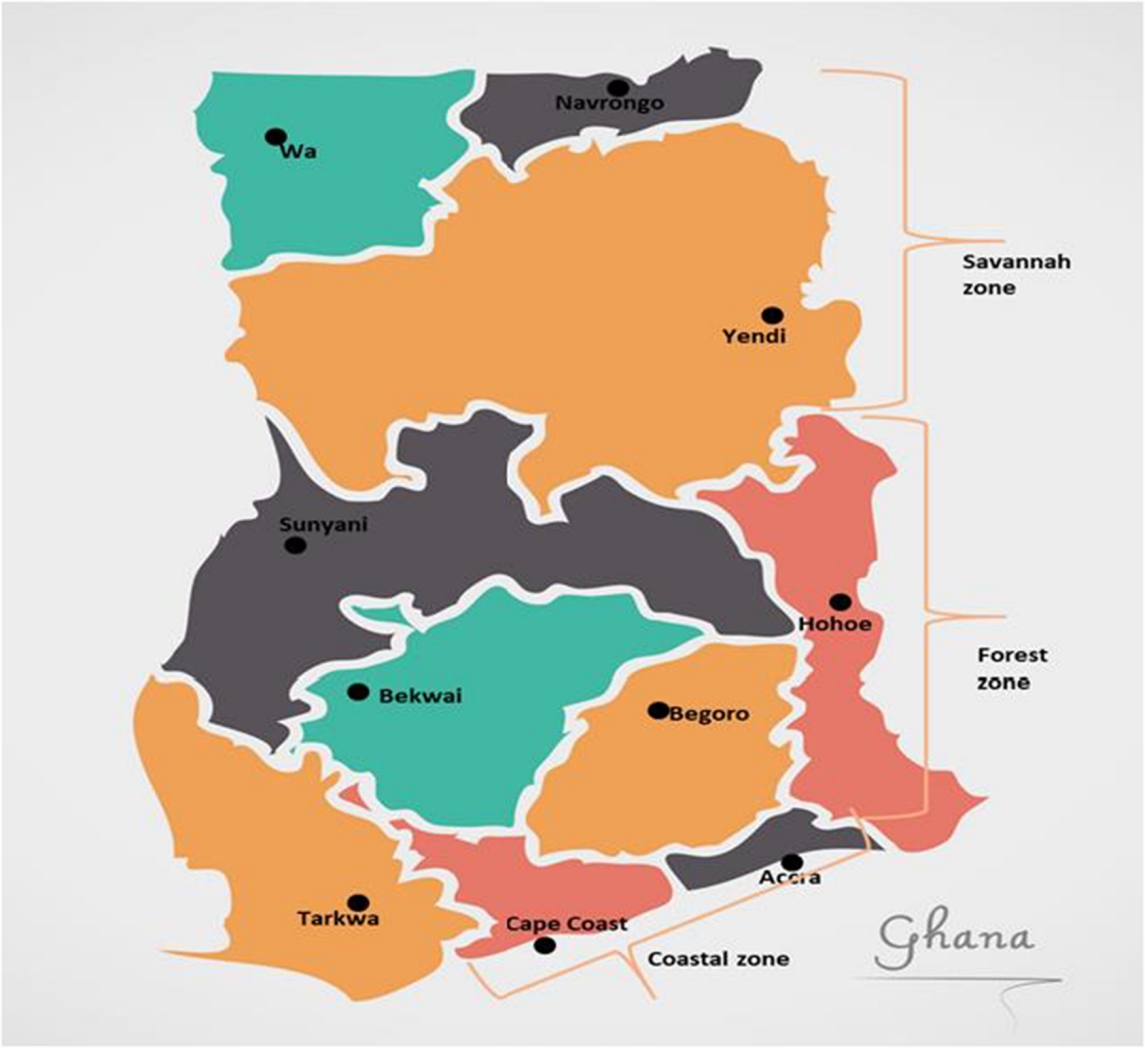
Map of Ghana showing the location of the sentinel sites in the three ecological zones. The sentinel sites for monitoring antimalarial drug efficacy/resistance in Ghana are in the 10 regions of the country. These sites have hospitals and health centres for treatment efficacy studies in malaria and were set up by NMIMR in collaboration with the National Malaria Elimination Program (NMEP).

There are 10 sentinel sites established by the National Malaria Elimination Program (NMEP) in collaboration with the Noguchi Memorial Institute for Medical Research (NMIMR) for Treatment Efficacy Studies (TES) on antimalarial drugs in Ghana (25)**.** The sentinel sites are located in three ecological zones of the country, Accra and Cape Coast in the coastal savannah zone (CS); Begoro, Bekwai, Hohoe, Sunyani, and Tarkwa in the forest zone (FZ); Navrongo, Wa, and Yendi in the Guinea savannah zone (GS) (Figure 1). Archived samples taken from children aged 9 years and below with uncomplicated malaria reporting to health facilities at the sentinel sites were used for this study (25). The samples were taken in the transmission seasons of time points, 2007–2008, 2010–2012, 2013–2014, 2015–2016, and 2018.

### Laboratory molecular analysis (PCR and Sanger sequencing)

Parasite DNA was extracted from filter paper blood blots using QIAamp DNA minikit (QIAGEN, Hilden Germany) following the manufacturer's protocol. Polymerase chain reaction (PCR) was carried out to amplify all the genes of interest for this study (*pffp2, pfcoronin, pfnfs, pffd, pfarps10, pfmrp1, pfmrp2, pfdhfr, and pfdhps*). The references of the protocols used, primer sequences, and cycling conditions for all the genes are shown in Supplementary Table S1.

For *pffp2*, the primary reaction was in a total volume of 15 µl and the secondary reaction was done in 50 µl with final reagent concentrations of 160 mM deoxynucleoside triphosphates (dNTPs), 160 nM primers, 1U Taq DNA Polymerase and 1X standard Taq Buffer [20 mM Tris-HCl (pH 8.9,), 1.8 mM MgCl_2_, 22 mM NH_4_Cl, 22 mM KCl, 0.06% IGEPAL CA-630, and 0.05% Tween 20] and 0.5 µl of genomic DNA. Amplification of *pffd* and *pfarps10* was conducted in a 30 µl reaction volume consisting of 1X PCR reaction buffer, 4 mM of MgCl2, 0.4 μM of each of the primers, 0.2 mM of dNTPs and 1 U of Taq polymerase. For *pfcoronin* and *pfnfs* amplification, a 25 μl reaction volume contained 12.5 μl 2X Kapa Hifi Hotstart ready master mix (Roche, SA) and 0.2 µM, primers (forward and reverse for *pfcoronin* and *pfnfs*) were used (Eurofins Genomics). The amplification of *pfmrp1* and *pfmrp2* was done in a total volume of 25 µl containing 0.2 µM of each primer, 0.2 mM deoxynucleotides triphosphate (dNTPs), and 2U One-Taq polymerase (New England Biolabs, Massachusetts, USA). Nested PCR was used to amplify the *pfdhps* gene with a primary amplification in 10 μl reaction mixture consisting of 0.4 μM of each primer and 5 μl 2X Amplitaq Gold DNA polymerase (Applied Biosystems; Thermofisher Scientific) master mix. 1 μl of the template was used in a 30 μl secondary reaction of 0.4 μM primers and 15 μl 2X Amplitaq Gold DNA polymerase. The *pfdhfr* was amplified in a single PCR run containing 0.4 μM of each primer and 15 μl 2X Amplitaq Gold DNA polymerase in a total volume of 30 μl. PCR products were detected using agarose gels stained with Gel red dye (Biotium, California, USA) or SYBR safe (Invitrogen, USA).

All Amplicons from the PCRs of the various genes were sequenced at Macrogen Europe (Amsterdam, Netherlands).

### Data analysis

There were variable sample sizes analyzed for each gene due to the availability of archived samples and funds, therefore computational analysis was done to determine haplotype and nucleotide diversities for *pffp2* only with samples for four time points. The chi-squared test for trend analysis was used to determine the trend of the prevalence of the *pffp2* mutations over the time points with StatCalc in Epi Info^TM^ 7.2.5.0 (Centres for Disease Control and Prevention, Atlanta, USA). Sequences were verified using the Basic Local Alignment Search Tool (BLAST) (http://blast.ncbi.nlm.nih.gov/) upon receipt from Macrogen Europe. This was used to determine the authenticity of the sequences of the genes. Multiple sequences were aligned with MAFFT (EMBL.EBI, Hinxton, Cambridge, UK) using the 3D7 wild-type sequences as references: *pffp2 *-* *PF3D7_1115700; *pfarps*10* *-* *PF3D7_1460900; *pffd *-* *PF3D7_1318100; *pfdhps *-* *PF3D7_0810800; *pfdhfr *-* *PF3D7_0417200; *pfnfs *-* *PF3D7_0727200; *pfmrp1 *-* *PF3D7_0112200; *pfmrp2 *-* *PF3D7_1229100; *pfcoronin *-* *PF3D7_1251200. Consensus sequence editing and single nucleotide polymorphisms (SNP) determination were carried out using the CLC main workbench 7.9.1 (Qiagen) and Benchling.com (California, CA, USA). Computational methods were employed for the *pffp2* gene to determine haplotype and nucleotide diversities. Base-calling, alignment, and deconvolution of Sanger chromatogram trace files were done using the command-line version of the application Tracy. The output binary variant call format (bcf) files for each sample were converted to human-readable variant call format (vcf) files using custom bash scripts. Low-quality variants (<40) and indels were filtered out from the vcf file after which the files were merged and variants extracted and annotated into a text file using custom bash scripts, SnpEff (v4.1), and vcf tools. Fasta files were generated using custom bash scripts and fed into DnaSP6.0 to determine the DNA polymorphism metrics and Tajima's D.

## Results

### Demographic data on study participants

**Figure 2:**
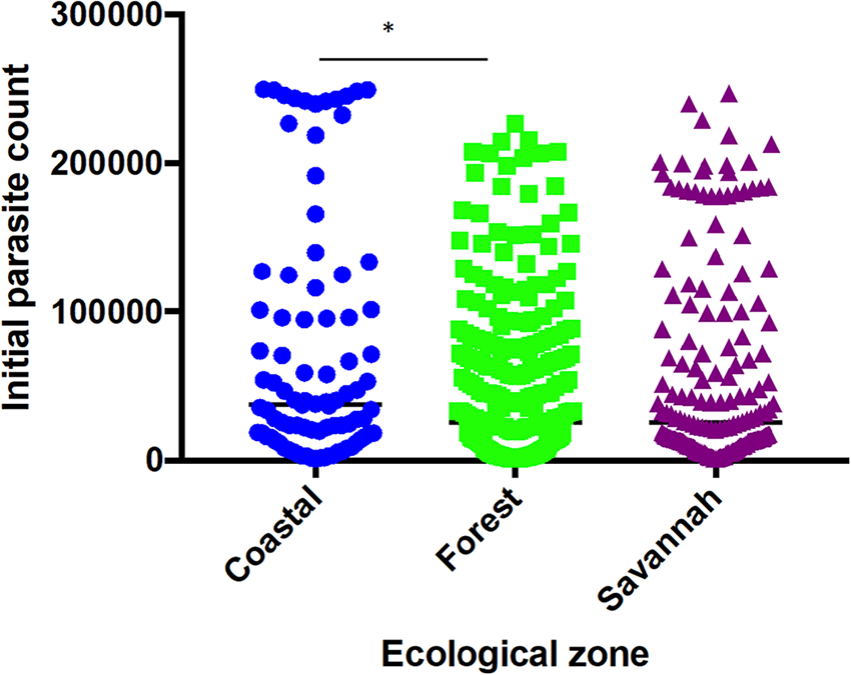
Parasite densities of study participants at the sentinel sites located in the three ecological zones. Each dot represents the parasite density determined per patient**.** There was a significant difference in parasite densities of participants from the coastal and forest zones, *p* ≤ 0.05 (*) using Dunn's test.

A total of 1,170 filter paper blood blot samples obtained from children aged ≤ 9 years with uncomplicated *P. falciparum* malaria were used for the study. The study population median age was 50 months (4 years and 2 months). The minimum parasite density observed among the study participants was 1,000 parasites/µl of blood and was recorded from the FZ and the maximum of 249,960 parasites µl/ of blood from the CS. There was a significant difference between the parasite counts from the CS and FZ (*P* ≤ 0.05) only (Figure 2).

### Pffp2

#### Reported and novel polymorphisms of *pffp2* in parasites from Ghana

**Figure 3:**
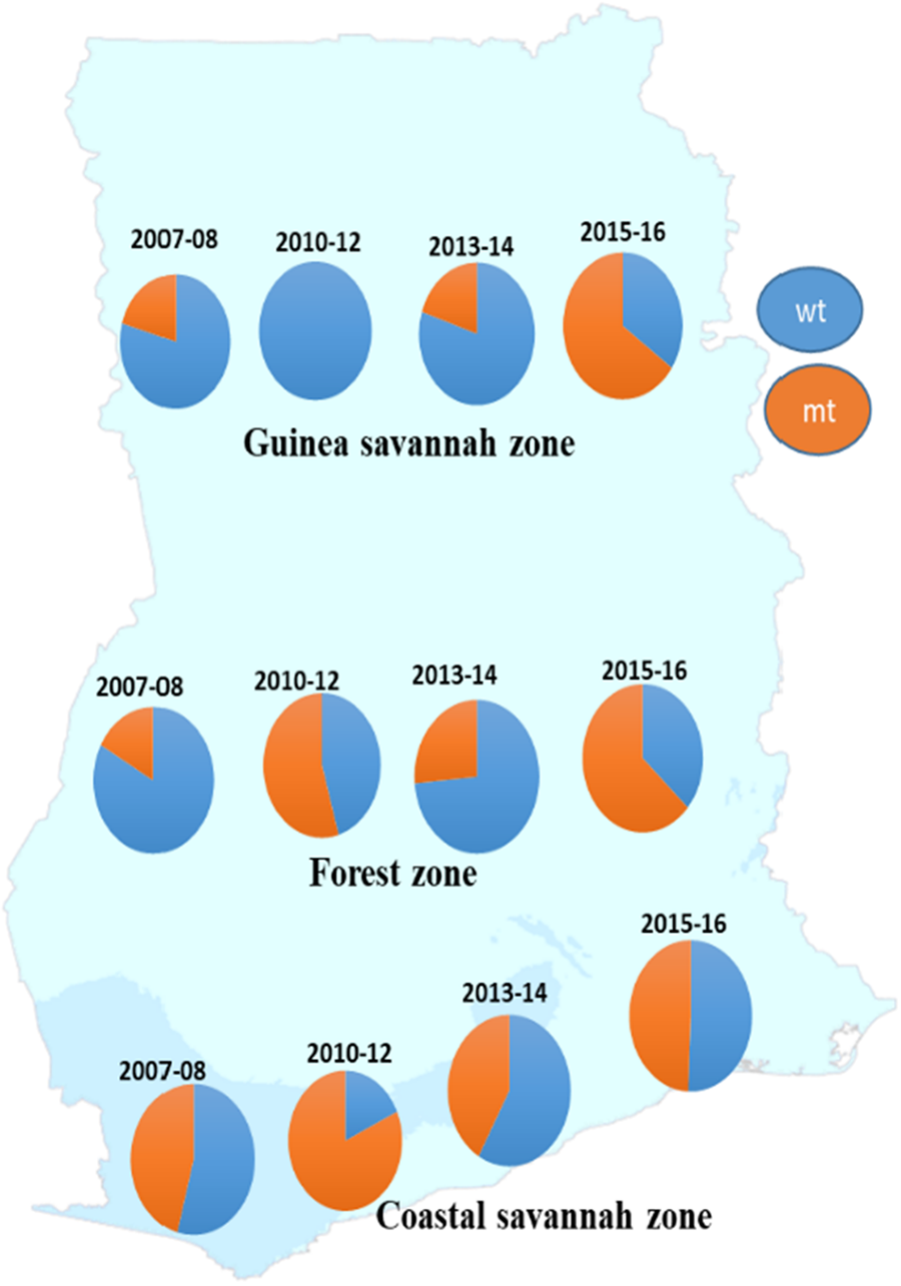
Distribution of the prevalence of *pffp2* wild-type and mutant alleles in parasite populations in the ecological zones at four time points. The prevalence of the wildtype and mutant alleles for the pffp2 varied with time and the three ecological zones, however, the timepoint 2015–16 showed the highest prevalence of the mutation for the FZ and GS.

**Figure 4:**
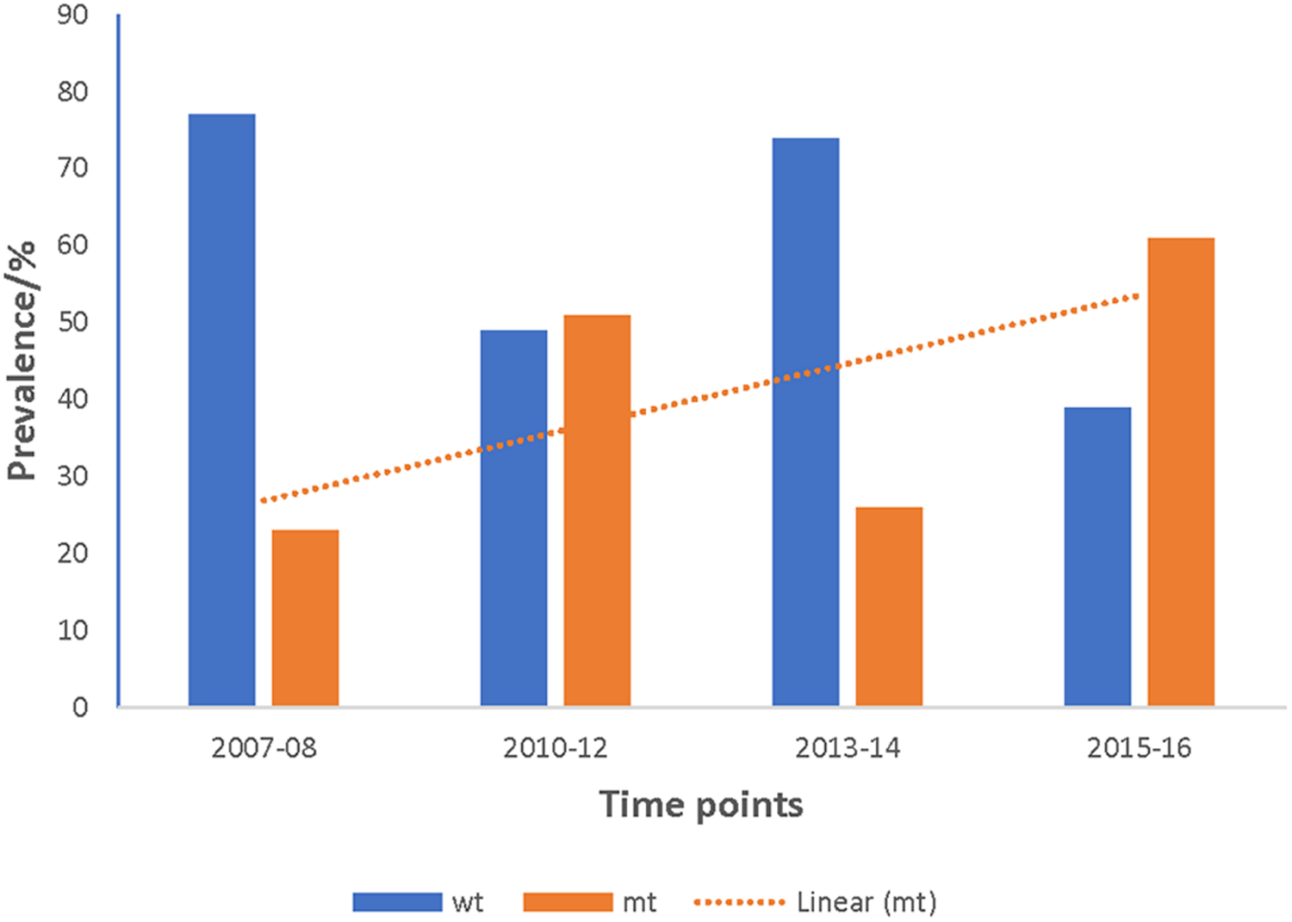
Increasing trend of the prevalence of *pffp2* mutant alleles in Ghanaian isolates over the four time points. The pooled data from all the sites for the four time points showed an increasing trend for the *pffp2* mutant alleles using the chi-square test for trends (*χ*^2^ = 16.7647, *p* = 0.00004).

A total of 872 good sequences were analyzed for polymorphisms in the *pffp2* and the proportion of mutant genotypes was 44.95% (392/872) of the isolates. The number of samples analyzed for each ecological zone and the proportion of mutants are shown in Table 1. Of the 392 mutants, there were 575 mutations with 81.6% (469/575) being non-synonymous (NS) mutations. Samples from time point, 2007–2008, showed 100 mutations at 110 codons, 2010–2012 had 28 mutations at 39 codons, 2013–2014 had 62 mutations at 68 codons, and 2015–2016 had 450 mutations at 245 codons. Mutations varied from a single mutation per codon to ten mutations per codon. Some of the mutations at specific codons were observed at various time points. The amino acid variants observed at codon 114 ranged from 1 to 10. The S35* mutation implicated in slow parasite clearance was not observed, however, five mutant variants, S35l/N/P/S/T, were observed. The synonymous S35S was observed in 19 samples. The frequency of other relevant mutations at codon 59 was 4.5% (26/575), codon 68 was 1.6% (9/575), and codon 69 was 0.9% (5/575). Other reported mutations implicated in ART resistance, V51I, S59F, S69P, and V70F were observed in 32.6% (128/392), 6.6% (26/392), 0.3% (1/392), and 0.5% (2/392) respectively of the mutants. Other mutations A8A, R27K, N32K, V21V, S43S, V47V, and V51I were observed in samples from all the ecological zones. Some zone-specific mutations observed were: GS: Y3D, M5l, Q16K/R, V21F/G, S43T, R60I, V70I, and K225Q; FZ: Y3N, D6N, H10Q/N, S14F, S35l, V51F, T56A/S/F, T65S, S68F, M167I, Y207F and H220l/P; CS: A8I, P9P, T56P, S68P, S69P, K255G, F260S, and R301K. For the time points, the polymorphisms, V51I and S59F were highest among the 2015–2016 samples and also observed at all the four time points. Others such as D151E were only observed in samples from 2010 to 2012, 2013–2014, and 2015–2016 whereas K255R was observed in parasite isolates from 2015 to 2016 only. The highest prevalence of mutations was observed in 2010–2012 at 81.8% for CS, and for the FZ in 2015–2016 at 63.4% whilst GS had the highest prevalence in 2015–2016 and was 65.3% (Figure 3). The prevalence of *pffp2* mutations showed an increasing trend over the time points (*χ*^2^ = 16.7647, *p* = 0.00004) as shown in Figure 4. All the observed *pffp2* mutations are listed in Supplementary Table S2.

**Table 1:** Number of samples per ecological zone at the time points and the proportion with *pffp2* mutations.

Zone	Year of sample collection					% mutants
	2007–08	2010–2012	2013–14	2015–16	Total	
CS	35	22	24	79	160	52.0
FZ	106	22	91	257	476	46.0
GS	68	15	55	98	236	38.0
Total	209	59	170	434	872	

Comparatively, the *pffp2* mutations were predominant in the isolates from the CS.

#### *Pffp2* haplotypes linked to delayed parasite clearance in Ghanaian malaria parasites

Haplotypes of the *pffp2* linked to delayed clearance in the SEA (26). were observed in Ghanaian isolates at various time points and are shown in Table 2. These include the mutant haplotype V47-I51 (VI) which was seen in isolates from all time points and the highest prevalence was 31.7% (126/398) and mostly in samples from the 2015–2016 time point. Other mutant haplotypes S105-K107-N108 (SKN) were not seen in samples from the 2013–2014 time point but were observed at the other time points. For K255-F260-Y265-D266 (KFYD) it was observed only in the 2007–2008 and 2015–2016 time points (Table 2).

**Table 2:** Observed *pffp2* haplotypes linked to delayed clearance in Ghanaian isolates.

Haplotype			Proportions with mutant haplotypes/%			
Wild-type (codons)	Mutant	*n*	2007–08	2010–12	2013–14	2015–16
V47-V51	VI	126	8.7	4.8	10.3	76.1
S105-K107-N108	NMK	14	29.0	14.0	0	57.0
K255-F260-Y265-D266	RFYD	25	32.0	0	0	68.0

*n*, number of samples with mutant haplotypes. These haplotypes were selected based on their link to slow parasite clearance reported elsewhere (Siddiqui et al. 2018).

#### *Pffp2* gene under purifying selection in Ghanaian malaria parasite population

Analysis for the distribution of SNPs across the genome showed that 2.4% were found in the intergenic region, 13.2% were missense variants, 29.4% were non-coding transcript variants, 9.7% were synonymous variants and 6.4% were frameshift variants (Supplementary Figure S1). The biological effect of the SNPs analyzed resulted mostly in missense variants. The time point 2015–2016 gave the highest number of haplotypes of 150 with 826 segregating sites. The FZ also gave the highest haplotype of 172 with 1116 segregating sites. Nucleotide diversity was low within the *pffp2*. The negative values for Tajima's D indicate purifying or negative selection. The mutation rates and the number of haplotypes observed within the *pffp2* for the ecological zones and time points are shown in Table 3.

**Table 3:** Mutation rate in the *pffp2* at the ecological zones and time points.

Zone	*π*	*S*	Tajima's D	Tajima's D (*p*-value)	No. of haplotypes/haplotype diversity (h/Hd)
CS	0.0325	421	−1.67274	*P* > 0.05	45 (1.000)
FZ	0.03773	1,116	−2.55376	*P* < 0.001	172 (0.9997)
GS	0.0348	928	−2.50291	*P* < 0.001	119 (0.9992)
Time point					
2007–08	0.05817	954	−2.21706	*P* < 0.01	78 (1.000)
2010–12	0.03201	622	−2.30326	*P* < 0.01	60 (0.9930)
2013–14	0.05453	751	−2.20532	*P* < 0.01	39 (1.000)
2015–16	0.02212	826	−2.58479	*P* < 0.001	150 (0.9993)

*S*; number of segregating sites, *π*; nucleotide diversity, D; Tajima's D. Tajima's D: *p* < 0.001, h; haplotype, Hd; haplotype diversity.

### Pfcoronin

#### Both known and novel *pfcoronin* polymorphisms in malaria parasites from Ghana

Ninety-six samples including post-treatment recurrent parasites collected in 2018 were amplified and sequenced for *pfcoronin*. Of the 90 good sequences analyzed, 53 (58.9%) were wildtype sequences and the mutants were 37 (41.1%). The number of mutations seen in the 37 mutants was 90, of which the majority were NS mutations (77.8%, 70/90). Of the 90 samples, the number of samples from the FZ, CS, and GS were 22, 19, and 49 respectively and the prevalence of mutants was 18% (4/22), 52.3% (10/19), and 46.9% (23/49) in the same order. The proportions of the 90 mutations observed in the samples were distributed in the zones as 6.7% (6/90), 24.4 (22/90), and 68.9% (62/90) for FZ, CS, and GS respectively. The most prevalent mutation was P76S observed in 24% (22/90: 6* *-* *CS; 2* *-* *FZ; 14* *-* *GS) of the isolates. It is a published mutation but with no link to ACT delayed clearance and characterized by a nucleotide base change from CCC-TCC. The known mutations, G50E, R100K, and E107V, linked to delayed parasite clearance with ACT use were not observed in the Ghanaian isolates. However, a variant of R100K which is R100R (AGA-AGG) was observed in only one isolate in the CS. The variants of some other published mutations, V62M, N112D/N/Y, and V128V were observed as; V62l, N112K, and V128E respectively. For pre-treatment and post-treatment isolates, 38.9% (35/90) and 61.1% (55/90) were mutants respectively. Statistical analysis showed a significant difference in the occurrence of SNPs between pre-treatment and post-treatment isolates at the three ecological zones (*p* = 0.00003). The mutations observed in the *pfcoronin* gene of all the isolates are shown in Supplementary Table S3.

### Pfnfs

#### *Pfnfs* polymorphisms associated with lumefantrine delayed clearance resistance in malaria parasites from Ghana

**Figure 5:**
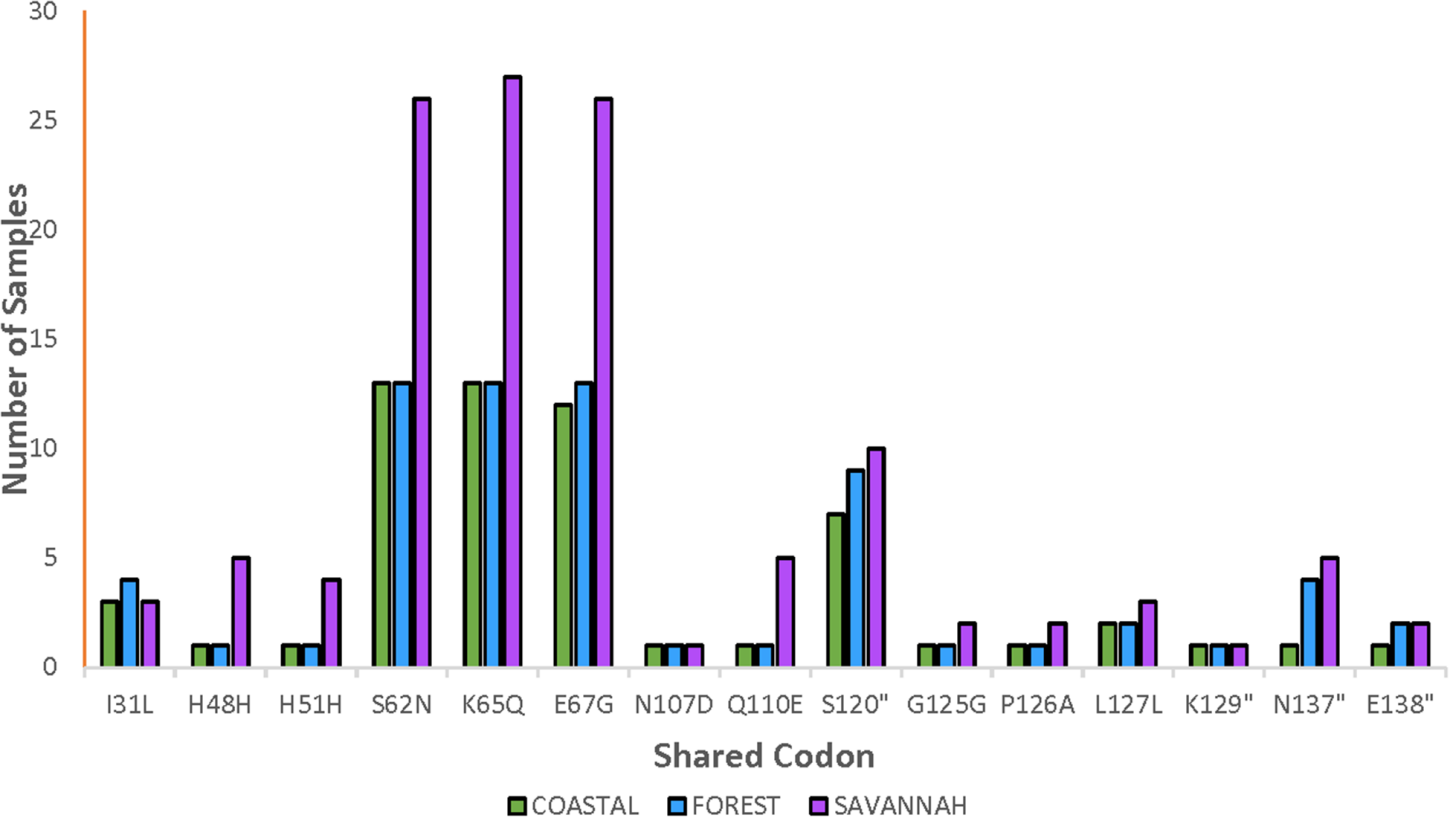
Distribution of shared *pfnfs* mutations in the three ecological zones. Some observed mutations of the *pfnfs* were seen in isolates from the ecological zones and were termed as shared mutations and are being displayed with the number of samples they were observed in at the three zones.

The total number of samples analyzed for the *pfnfs* was 96 samples collected in 2018 which includes recurrent samples. Ninety-two good-quality sequences were analyzed for the identification of mutations, of which 26.1% (24/92) were wild-type genotypes. The 92 samples are distributed as 22, 20, and 50 for the FZ, CS, and GS respectively. The 68 mutants had a total of 356 mutations and the majority were NS (88.2%, 314/356). Of the 356 mutations, 62.1% (221/356) SNPs were detected in the pre-treatment samples and 37.9% (135/356) in the post-treatment samples. The highest occurring mutation, K65Q, was 14.9% (53/356) of all mutations observed followed by S62N in 14.6% (52/356) and E67G in 14.3% (51/356). These are the three known SNPs linked to delayed parasite clearance for lumefantrine. The shared and most dominant mutations are shown in Figure 5. Analysis of the proportion of pre-treatment and post-treatment SNPs from the three ecological zones revealed no significant difference (*p* = 0.76). There were some mutations shared between both the pre-treatment and post-treatment samples. The K65Q occurred the most in the pre-treatment samples with a prevalence of 15.4%. It is interesting to note that this mutation together with S62N and E67G had the same prevalence (14.1%) in the post-treatment samples.

### Pfarps10

#### Polymorphisms in *pfarps10* linked to ART resistance in parasites from Ghana

Ninety-five samples collected in 2018 were analyzed for the *pfarps10* for the detection of mutations. The wildtype parasites were 63.2% (60/95) of the isolates with ecological representations as 62.5% (20/32) from CS, 65.6% (21/32) from FZ, and 61.3% (19/31) from GS. Codon 127 had the highest number of variants, 13.7% (13/95) among the isolates. V127W was the highest occurring mutation observed in 6.3% (6/95) of the isolates followed by H126M in 5.3% (5/95) and then D128I in 4.2% (4/95). V127M is one of the PGB mutations of ART resistance and was seen in one sample from the GS. Other variants V127D, V127G, and V127C, were observed in one, two, and two samples respectively. The observed mutations in all isolates are listed in Supplementary Table S4.

### Pffd

#### None of the drug resistance polymorphisms of *pffd* observed in parasites from Ghana

A total of 96 samples collected in 2018 were sequenced of which 91 were of good quality and used for analysis. The proportion of wild-types in these samples was 91.2% (83/91). No mutation of this gene was observed in samples from the FZ, however, the CS and the GS zones had two and six mutants respectively. In all, 15 mutations were observed in the eight mutants and the majority were NS (73.3%, 11/15). V148G was the most prevalent mutation; occurring in 3 isolates. Apart from the shared mutations by all the zones, of the 15 mutations, 13 (86.7%) were from GS. SYN mutations recorded were V126V, V182V, T174T, and P177P. There was a change from lysine to a stop codon at codon 146 (K146*) in one isolate. Other observed mutations, A145S, E149K, K187N, D189H, M194V, D161Y, E162D, E163Y, Q164H, and K146* were from isolates from GS. The published marker, D193Y was not observed in the samples analysed. All observed *pffd* mutations in the isolates are shown in Supplementary Table S5.

### Pfmrp1

#### Polymorphisms of *pfmrp1* linked to drug resistance in parasites from Ghana

**Figure 6:**
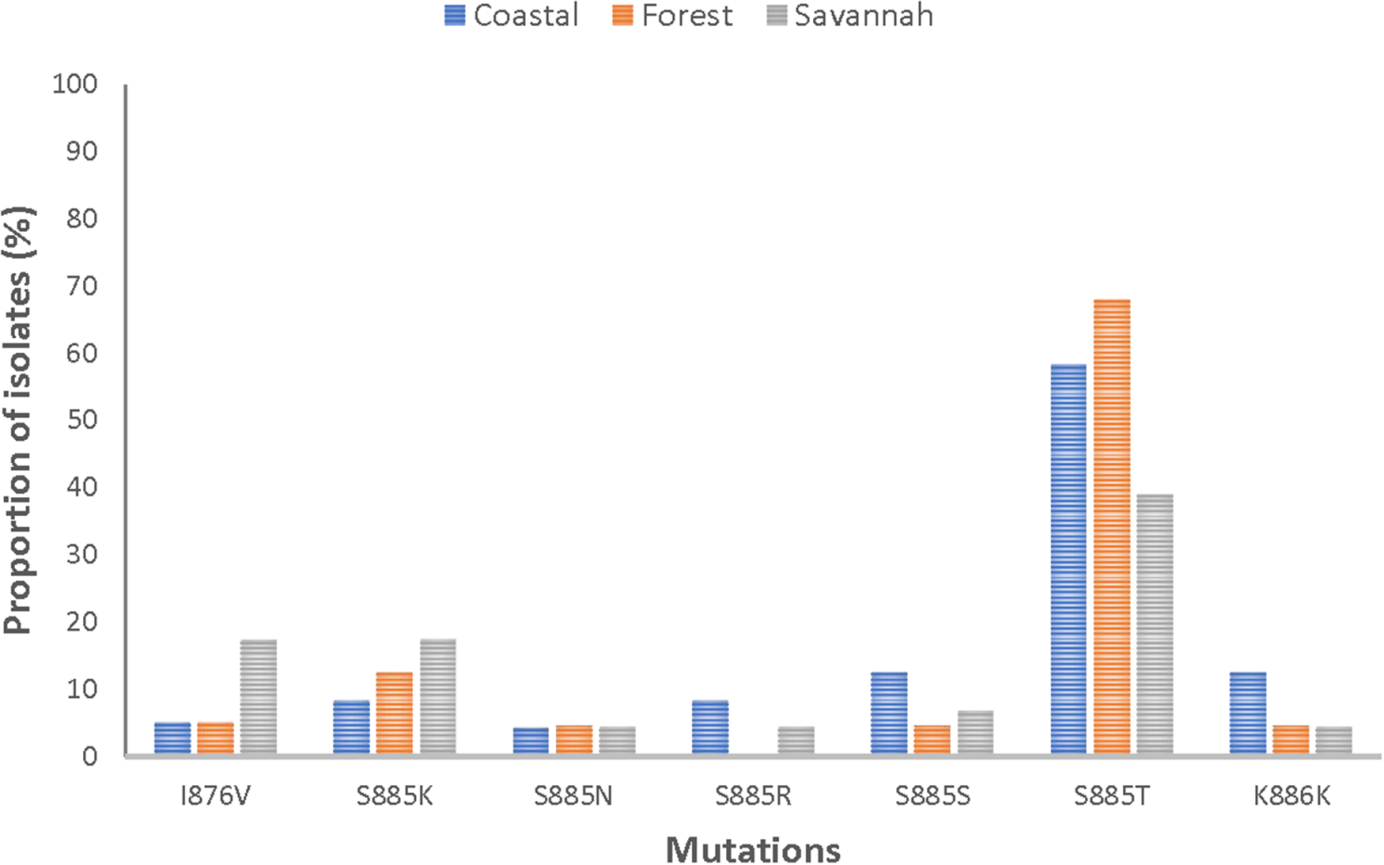
Proportion of isolates with shared *pfmrp1* mutations in the three ecological zones. Some of the observed mutations were seen in isolates from the three ecological zones and have been depicted as proportions of isolates they were observed in.

Of the 84 good-quality sequences, the proportion of wild-type was 4.8% (4/84). The 80 mutants had a total of 58 mutations, with 89.7% (52/58) being NS. It was observed that 75% (63/84) of the mutants had two or more mutations. The following mutations; I876V, S885K, S885N, S885S, S885T, and K886K were detected in all mutants. Figure 6 shows the distribution of the prominent mutations detected across the zones. At least one SNP was detected in 96.3% (26/27), 95.1% (39/41), and 93.8% (15/16) of the analyzed sequences from CS, FZ, and GS respectively. There was no statistical difference in the number of SNPs between the three zones (*p* = 0.620815). S885T was the most frequent mutation and was found in 63.1% (53/84) of the isolates and was highest in FZ with a prevalence of 78% (32/41). The I876V mutation which is reportedly linked to AL resistance was observed in in 9.5% (8/84) of the isolates. GS isolates had the highest number of this mutation 50% (4/8). All observed *pfmrp1* mutations in the isolates analyzed are shown in Supplementary Table S6.

### Pfmrp2

#### None of the polymorphisms of *pfmrp2* linked to drug resistance in parasites from Ghana

**Figure 7:**
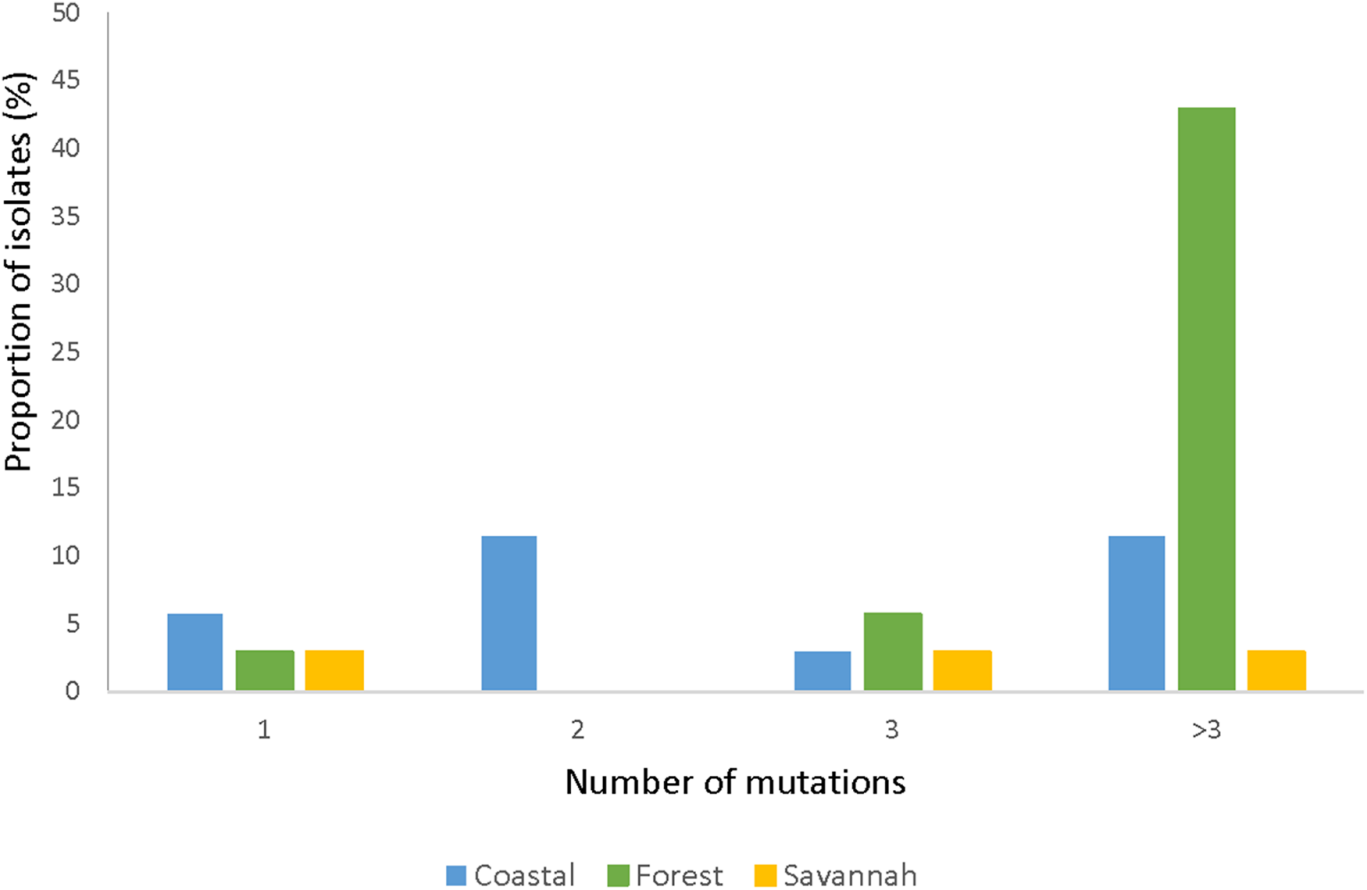
Proportion of isolates with single and multiple *pfmrp2* mutations in isolates from the ecological zones. For the *pfmrp2* mutations, it was observed that one isolate could have up to three mutations, and the proportion of such isolates at the three ecological zones is portrayed by the histogram.

The total number of samples analyzed for *pfmrp2* was 105. Thirty-five good sequences were analyzed. The prevalence of wild-type was 8.57% (3/35) and the 32 mutants had a total of 126 mutations, of which 94.4% (119/126) were SNPs and 5.6% (7/126) were insertions. Out of the 119 SNPs, 87.4% (104/119) were NS mutations with 2.9% (3/104) being nonsense mutations i.e., a change from AA to a stop codon. Of all the mutants observed, 87.5% (28/32) of the isolates had multiple mutations while 12.5% (4/32) had a single mutation. Figure 7 shows the prevalence of the single or multiple SNPs detected in samples from each ecological zone. The most abundant mutation detected was N645D (8.0%, 10/126). The SNPs D631G and K714I followed with a frequency of 5.5% (7/126) each. The most prevalent SYN mutation was P566P which was detected in 8.6% (3/35) of the isolates. None of the SNPs was shared across all the zones. SNPs detected multiple times in isolates from FZ are I533l, R534K, T550P, P566P, E574K, K600K, N622S, D629N, D633N, Y636C, D637N, D649N, M652V, and Q694H. The SNP N622D which was reported by Veiga and others was not detected, however a variant N622S was seen. All observed *pfmrp2* mutations in the isolates analyzed are shown in Supplementary Table S7.

### Pfdhfr

#### High prevalence of the triple mutation of *pfdhfr* in parasites from Ghana

**Figure 8:**
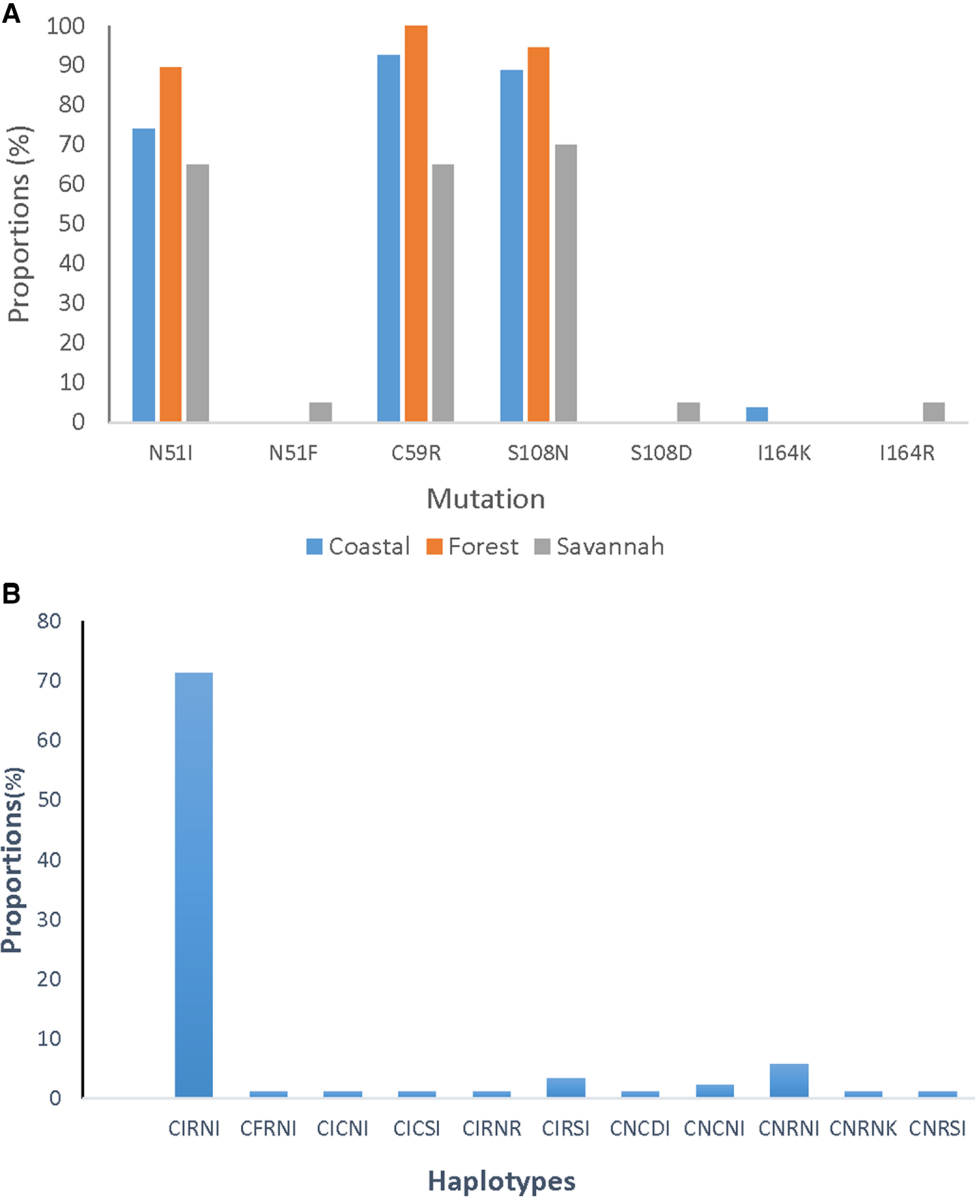
Proportion of *pfdhfr* mutations and haplotypes from Ghana. (**A**) The proportion of isolates with the mutations I51, R59, and N108 are still dominating in the parasites but were seen as highest in the isolates from the FZ followed by CS and GS. (**B**) The CIRNI haplotype which is a mutant genotype still dominates in the parasite population in Ghana with a prevalence above 70%.

A total of 85 good sequences were analyzed for the *pfdhfr* gene with 99% (84/85) having mutations. The known mutations N51I, C59R, and S108N were observed in a majority of the samples. C59R was observed in 89.4% (76/85), S108N in 87.1% (74/85), and N51I in 78.8% (67/85) of the isolates. Two rare variants, N51F and S108D were observed in one parasite isolate from GS. Rare variants of the I164l occurred in isolates, I164R from GS and I164K from CS. The most prevalent haplotype for the *pfdhfr* gene was the triple mutant IRN observed in 72.9% (62/85) of the isolates. The double mutant RN haplotype was also observed in 5.9% (5/85) of the isolates. The proportions of *pfdhfr* mutations and haplotypes in the isolates for the ecological zones are shown in Figure 8.

### Pfdhps

#### A new mutation, K540N of *pfdhps,* with high prevalence in parasites from Ghana

**Figure 9:**
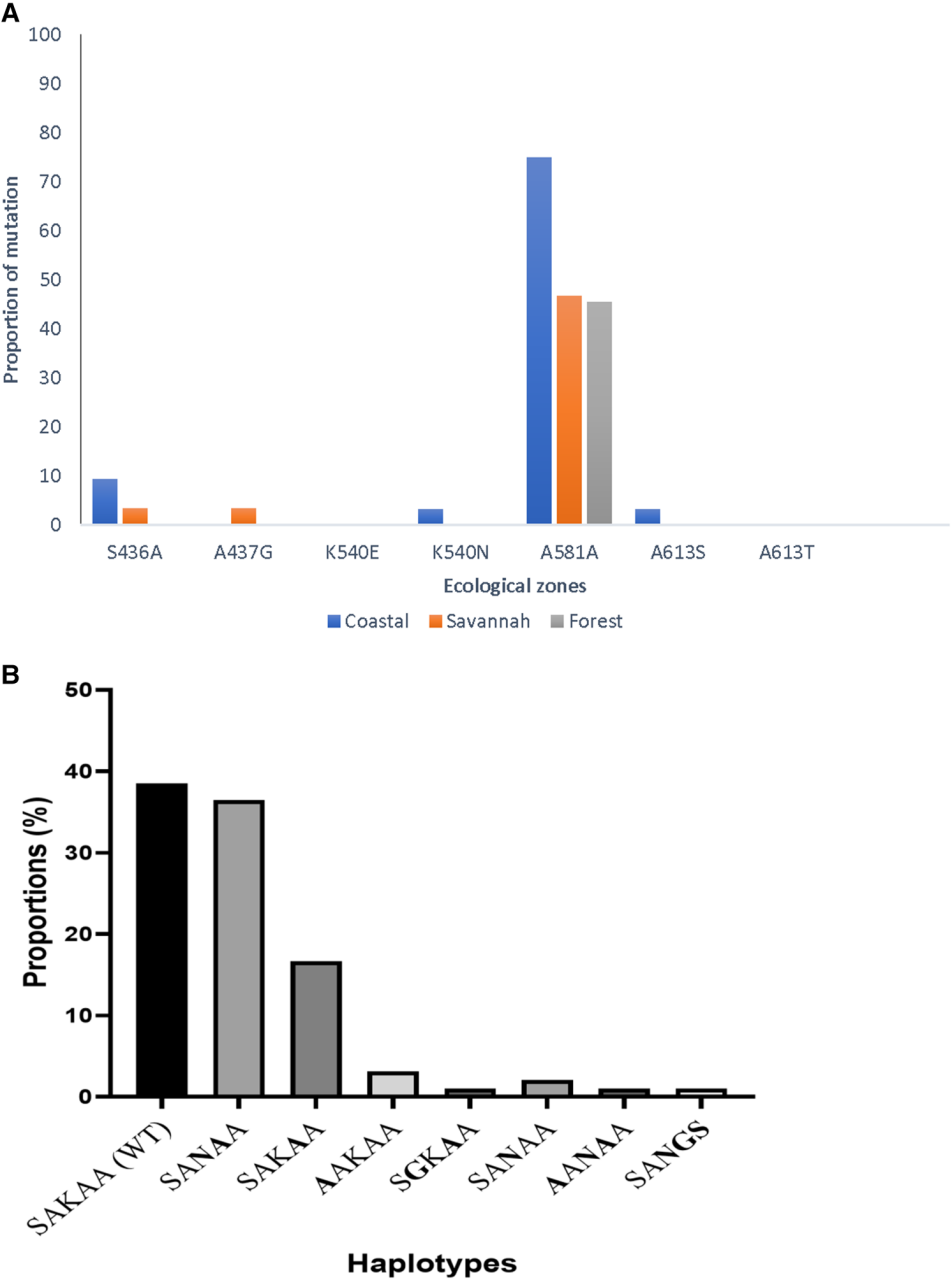
Proportion of *pfdhps* mutations and haplotypes in isolates from Ghana. (**A**) The proportion of isolates with both known and novel *pfdhps* mutations are shown. The K540E mutant for sulphadoxine resistance was absent, however, a new synonymous mutation A581A was seen to predominate in isolates from all the ecological zones. (**B**) The wild-type haplotype, SAKAA, predominated the mutant haplotypes observed in the isolates. The amino acids in **bold** are the SNPs.

In all, 95 good sequences were analyzed for the *pfdhps* and 72% (68/95) had mutations. The most observed mutation was A581A (GCG to GCA) in 78.0% (53/68), followed by K540N at 57.3% (39/68) of the mutants. The aforementioned mutations are variants of A581G and K540E associated with sulphadoxine resistance, however, the K540E was not observed in the Ghanaian samples. There was also no detection of the novel I431V mutation, seen in pregnant women from Cameroon and Nigeria in the Ghanaian isolates. The proportion of isolates with the different mutations from the three zones as well as the haplotypes are shown in Figure 9. There were zone-specific mutations observed such that S436A was observed in 4.2% (4/95) of the samples from FZ (3) and GS (1). In addition, A437G was only observed in 1.1% (1/95) of the isolates from GS.

In summary, all the genetic mutations of known relevance in ACT and SP resistance as well as novel mutations in the nine genes investigated in the Ghanaian *P. falciparum* isolates from symptomatic patients are shown in Table 4.

**Table 4:** Summary of observed markers of antimalarial drug resistance and variants in malaria parasites from Ghana.

Gene	Drug affected	Published molecular markers	References	Observed markers and variants in Ghanaian isolates	Some observed novel mutations in Ghanaian isolates
*pffp2*	Artemisinin and derivatives	N4H, S35*, V51I, S59F, S69*, K255R	(26–28)	** N4H**, S35l/P/T, **V51I**/F, **S59F**, S69S/P, **K255R**/G	E18K, K31K, N32K, V47V, R60K, I79K, N108K, D151E
*pfcoronin*	Artemisinin and derivatives	G50E, V62M, R100K, E107V, N112N/Y/D	(12, 29–32)	V62l, R100R, N112K	P76S
*pfnfs*	Lumefantrine	S62N, K65Q, E67G	(17, 28)	** S62N**, **K65Q**, **E67G/**D	Q110E, S120I
*pfarps10*	Artemisinin and derivatives	V127M	(16, 28, 33)	V127C/D/G/W	–
*pffd*	Artemisinin and derivatives	D193Y	(16, 28)	–	–
*pfmrp1*	Sulphadoxine-pyrimethamine, Chloroquine, Quinine	H191Y, S437A, I876V	(18, 34, 35)	** I876V**	M863Y, D864G, D865*, L862S, N866Y, S885T
*pfmrp2*	Chloroquine, Quinine, Mefloquine	N622D	(19)	–	D631G, N645D, K714I
*pfdhps*	Sulphadoxine	A437G, K540E, A581G, A613S	(21, 36–40)	** A437G**, K540N, **A581G**/A, **A613S**/T	N521K, H614l
*pfdhfr*	Pyrimethamine	N51I, C59R, S108N, I164l	(39, 41–44)	** N51I**/F, **C59R**, **S108N**/D, I164R/K	D54N, R106K, E134K, D139N, N230K

*, stop codon; –, not observed; bold, observed published molecular markers in Ghanaian isolates.

## Discussion

Evolving antimalarial drug resistance in the malaria parasite, especially for the ACT regimens which are currently characterized by slow parasite clearance, is of great concern because it threatens malaria elimination at the country level as well as the global disease eradication plan. One important recommendation for guaranteeing country-level disease elimination is increasing molecular surveillance of the parasite population in disease-endemic areas which involves monitoring the prevalence of molecular markers of antimalaria drug resistance. These markers are polymorphisms occurring in genes encoding important proteins for multiple cellular and metabolic processes for parasite survival. The findings from this investigation revealed numerous polymorphisms in the nine genes studied (*pffp2, pfcoronin, pfnfs, pfarps10, pffd, pfmrp1, pfmrp2, pfdhfr, and pfdhps*), and most of the known markers of drug resistance were identified in the Ghanaian isolates. Variants of some of the known markers of antimalarial drug resistance were also observed. One of the genes (*pffp2)* investigated is under purifying selection and showed increasing trends in the prevalence of mutations over the years.

Polymorphisms observed in the *pffp2* gene were diverse and the S69* mutation linked to slow parasite clearance by Ariey and others (4) was not observed in our study. This observation is similar to studies carried out in Uganda and Kenya (27, 28). The mutations, N4H, V51I, and S59F, also linked to slow parasite clearance (26, 27) were observed in our isolates and occurred in isolates from the three ecological zones. The study in Kenya also reported the observation of these mutations, S59F, N4H, A8I, P9P, H10N, and E11E as seen in the Ghanaian isolates (28). Most of the *pffp2* mutations were shared among the isolates from the different ecological zones, signifying similar selective forces such as host immunity and drug pressure (Zhu et al. 2016). The HIF (15-51-59) haplotype observed in this study has been seen in isolates from the SEA region (26). The parasites with the HIF haplotype were reported as having a high Michaelis–Menten constant (Km) which implies that a higher substrate (Z-LR-AMC, a preferred substrate for falcipain 2) concentration was needed to enhance enzyme activity (26). The observation of the increasing prevalence of *pffp2* mutations in the circulating parasite population could be indicative of emerging ART-resistant parasites in Ghana and may contribute to the 5% decrease in the efficacy of both AS-AQ and 3% for DHAP (25, 45). The nucleotide diversity (*π*) in the *pffp2* gene was very low which suggests a decreased probability for selection in the gene. The estimated negative Tajima's D values may also be suggestive of a recent population expansion with multiple low-frequency variants.

The *pfcoronin* mutations, G50E, R100K, and E107V, which have been associated with reduced *P. falciparum* susceptibility to ART (12), were not observed in both pre-treatment and post-treatment isolates. However, a SYN variant R100R of the R100K was observed in an isolate from CS. The mutation, P76S, was seen in isolates from all three ecological zones at a prevalence of 24%, which is higher than those observed in studies conducted in Senegal, Gabon, Ghana, Kenya, and Congo, where the prevalence ranged from 4% to 17% (29, 30). The observation of mutations in this study is not in accordance with reports from Senegal and Pakistan where no *pfcoronin* mutants were observed (46, 47). The *pfcoronin* mutations in the pre and post-treatment isolates had equal proportions of the known SNP P76S. The same samples were used for the detection of mutations in *pfnfs* and the K65Q mutation was the most dominant mutation (14.9%) which occurred in isolates from all three zones. In addition, the S62N (14.6%) and E67G (14.3%) were also observed which is similar to reports from a study by Wamae and others (2019), where the prevalence of mutations was reported to be high. In that study, the K65Q was in high linkage disequilibrium with S62N and E67G (28). The *pfnfs* mutation K65Q, was more prevalent in the pre-treatment than post-treatment isolates.

The *pffd* gene forms part of the PGB associated with *pfk13* mutations in enhancing ART resistance. In this study, the D193Y associated with slow parasite clearance (16) was not observed in the isolates which is contrary to a report from Sudan where 46.3% of isolates carried this mutation (48). The majority of the isolates studied were wild-type genotypes (91.2%) which is supportive of the low rate of spontaneous mutations in *pffd* as reported in a study in Kenya (28). The *pfarps10* mutation, V127M implicated in ART resistance (Miotto et al. 2015), and other variants (V127C/D/G/W/*) were detected but at low frequencies. The V127M was observed in one sample just as reported by a Ugandan study (33), however, another study in Asia reported observing the mutation in 41% of the isolates investigated (49). The Ugandan study also reported the D128H mutation which was detected in one isolate, however, its relevance to resistance remains unknown (33). Other variants of this SNP (D128G/I/K/N) were also observed but their relevance to drug resistance is yet to be investigated. The low prevalence of the *pfarps10* gene mutations that were identified in this study was in agreement with previously investigated PGB mutations, however, studies carried out in Kenya, Mali, and Tanzania observed no *pfarps10* mutations (28, 50, 51).

*Pfmrp1* and *pfmrp2* are 41% identical at the amino acid level and therefore have similar antimalarial drug targets (34, 52). The *pfmrp1* I876V mutation was present in 7.7% of the isolates and this is interesting because the mutant genotype has been reported to be highly selected under AL pressure in Tanzania and Zanzibar (53). Mutations at codon 885 were five unique variants (S885G/K/N/R/T) and these were observed in all three ecological zones at varied frequencies. This codon would be monitored in subsequent TES to detect the selection pressure and its relevance in drug resistance in the parasite population. The *pfmrp2* K714I observed by Veiga and colleagues in Thai isolates and reported to be linked to slow parasite clearance (19) was detected in Ghanaian isolates. Another mutation reported from the same study, N622D, was not observed in our samples, however a variant N622S was detected. There were several other *pfmrp2* novel mutations observed in our isolates which have not been linked to slow parasite clearance currently and need functional characterization.

The study showed a high prevalence of *P. falciparum* isolates with *pfdhfr* mutations (IRN) in Ghana as reported in previous studies over the years and is approaching saturation (7, 54, 55). Comparatively, the *pfdhps* mutations are in low prevalence with the key mutation, K540E for resistance to sulfadoxine still not detected in Ghanaian isolates. Interestingly, 41.1% of the isolates had a variant mutation K540N. The relevance of this mutation is yet to be ascertained. Nevertheless, this does not prevent the likelihood of sulfadoxine resistance in the population since some of the isolates had both S436A and A437G mutant alleles. This is similar to a study conducted in Burkina Faso, where none of the isolates carried the K540E mutation (56). The A581G and A613S mutants observed for failure of SP prophylaxis in pregnancy (7, 57) were also detected in this study. The *pfdhps* I431V mutation observed in pregnant women in Cameroon and Nigeria (36, 37) was not observed in any of the isolates which could be due to the small sample size compared to those used in the two previous studies.

The distribution of the mutations in the various genes investigated at the three ecological zones showed interesting patterns. It was detected that mutations observed in the *pffd*, *pfnfs*, *pfcoronin,* and *pff2* were highest in the GS, whilst those of *pfmrp2, pfdhps, pfdhfr,* and *pfmrp1* dominated in the FZ and *pfarps10* mutations were predominant in the CS. The observation of shared, zone-specific, and novel mutations in the ecological zones for these genes may be suggestive that malaria transmission intensity as well as human migratory patterns, may have played a role in the distribution of mutations (5, 58–60). Typically, areas with high transmission intensity show increased exchange of genetic material amongst the parasite population which subsequently leads to the development of spontaneous mutations that can confer resistance to antimalarial drugs (5, 61). In addition, the observation of both zone-specific and shared mutations across the zones may indicate gene flow amongst individuals in the population through intra-country migration (5, 6, 62).

## Conclusion

Broadly translated, our findings indicate that *P. falciparum* genes may be under drug pressure in the country following over a decade of use of ACT regimens and SP. Sustained drug pressure, aided by reduced sensitivity to ACT partner drugs may lead to strong selection for the *de novo* evolution of resistance in Africa. The observation of novel mutations requires further surveillance efforts to determine their roles in the tolerance or susceptibility of the Ghanaian *P. falciparum* populations to ACTs. Our data strongly supports the relevance of continuous molecular surveillance of markers of antimalarial drug resistance in Ghana and other malaria-endemic countries of the world to aid in the collation of evidence-based scientific data for global malaria eradication efforts.

## Data Availability

The datasets presented in this study can be found in online repositories. The names of the repository/repositories and accession number(s) can be found below: [https://www.ncbi.nlm.nih.gov/ from OR498143 to OR498204].
