# Supplementary material for: Putative molecular markers of Plasmodium falciparum resistance to antimalarial drugs in malaria parasites from Ghana
Source: Front Epidemiol. 2024 Feb 15;4:1279835. doi: 10.3389/fepid.2024.1279835 (PMC10910922; doi:10.3389/fepid.2024.1279835)
Supplement: Supplementary file 1 [file Datasheet1.pdf]

Supplementary Tables and Figure

Supplementary Table 1 Primer sequences, PCR conditions used for the amplification of the genes

Gene	Primer sequences '5-3'	Amplicon size (bp)	PCR cycling conditions	Reference
pffp2	1° F: TGTAGCAAGAACGTTTTGTGTAAAT R:GGTAAAGGAAAAATTAGTAAGGATGC	2029	94°C for 5 mins; 35 cycles of (94°C for 30s, 60°C for 60s, 68°C for 90s); 68°C for 10mins	**(Conrad et al., 2014)**
	2° F:TGTGTAAATTAAAGATAAAAGTGCAAA R: GGTCCCTTTTTAAAATACTATTGACA	1450	94°C for 5 mins; 35 cycles of (94°C for 30s, 60°C for 60s, 68°C for 90s); 68°C for 10mins	
pfarps10	F: CACAATATTATGTTTCATTTTAG R: GTATAATTTATTCTGCTTACATTC	165	95°C for 5 mins; 40 cycles of (95°C for 30s, 56°C for 45s, 68°C for 30s);72°C for 7mins	**(Wamae et al., 2019)**
pffd	F: GATGCTAGTGAAAGACAGAAT G R: CAC ATA TTT TTG ATT GAG GAC	194	95°C for 5 mins; 40 cycles of (95°C for 30s, 56°C for 45 sec, 68°C for 30 sec); 72°C for 7 min	**(Wamae et al., 2019)**
pfdhfr	F: TCCTTTTTATGATGGAACAAG R: AGTATATAC ATCCTAACAGA	700	95°C for 3 mins; 45 cycles (92°C for 30s, 45°C for 45s, 68°C for 45s); 68°C for 3mins	
pfdhps	1° F: AACCTAAACGTGCTGCTGTTCAA R: AATTGTGTGATTTGTCCACAA	711	95°C for 5 mins, 35 cycles of (95°C for 30s, 50°C for 30s, 68°C for 1 min); 68°C for 5 mins	**(Oguike et al., 2016)**
	2° F: ATGATAAATGAAGGTGCTAG R: TCATTTTGT TGTTCATCA TGT	647	95°C for 5 mins, 35 cycles (95°C for 30s, 52°C for 30s, 68°C for 1 min); 68°C for 5 mins	
pfcoronin	F: ATGTATAATGTTCCTTTAATCAAGA R: CTTTGGCATATTTATTTCAAAGG		95°C for 5 mins, 40 cycles of (95°C for 30s, 56°C for 45s, 68°C for 30s); 72°C for 7 mins	**(Demas et al., 2018)**

pfnfs	F: CTTCAATTTTGTAATGAAATTTCTTC<br>R: CATATTATGTCCATATAAACTTTGGA		95°C for 5 mins, 40 cycles of (95°C for 30s, 56°C for 45s, 68°C for 30s); 72°C for 7 mins	**(Wamae et al., 2019)**
pfmrp1	1º<br>F: AATATTCCATTCAATGAAAATTAC<br>R: CAACGTACTTTTATTCATTGAGA	208	95°C for 9 min, 45 cycles of (95°C for 40s, 50°C for 40s, 72°C for 40s); 72°C for 5 mins	**(Dahlstrom et al., 2009)**
	2º<br>F: TATTCCATTCAATGAAAATTACCT<br>R: TATGGAAGGATCTAAAGATGTAAA	109	95°C for 9 mins, 45 cycles of (95°C for 40s, 50°C for 40s, 72°C for 40s); 72°C for 5 mins	
pfmrp2	1º<br>F: ACAAGAATAAGTAATATGCATCATA<br>R: TAGATTCATCATACTTATTTCCG	1171	94°C for 2 min; 10 cycles of (93°C for 15s, 57°C for 30s, 72°C for 90s); 25 cycles of (93°C for 15s, 55°C for 30s, 72°C for 90s);<br>10 cycles of (93°C for 15s, 53°C for 30s, 72°C for 90s); 72°C for 10 min	**(Veiga et al., 2014)**
	2º<br>F: TGGGAATCTATAGCTTTTGAT<br>R: TGTGGTACATATAAAATAGGCA	1049	94°C for 2 min; 10 cycles of (93°C for 15 sec, 57°C for 30 sec, 72°C for 90 sec); 25 cycles of (93°C for 15 sec, 55°C for 30 sec, 72°C for 90 sec); 10 cycles of (93°C for 15 sec, 53°C for 30 sec, 72°C for 90 sec); 72°C for 10 min	

Supplementary Table 2 Observed *pffp2* mutations in all the Ghanaian isolates analyzed.

Mutation	FZ	GS	CS	Mutation	FZ	GS	CS	Mutation	FZ	GS	CS
Y3D	-	-	1	K89S	-	-	1	E160A	1	-	-
Y3N	-	1	-	K90I	-	1	-	E160K	1	-	-
N4H	2	-	3	K90N	1	1	3	H161N	1	-	-
M5R	1	8	5	F91I	1	-	-	H161R	1	-	-
M5I	-	1	-	I92N	-	-	2	H161Y	2	-	-
M5L	-	-	1	I92V	1	-	-	I162K	1	-	-
M5V	-	1	-	V93A	-	8	5	I162L	-	1	-
D6N	-	1	-	V93L	-	-	1	I162T	1	-	-
D6V	-	1	-	S94A	-	-	1	N163T	3	1	1
D6Y	-	2	-	S94P	-	4	2	Q164H	1	-	2
Y7Y	-	5	1	S94S	-	-	2	Q164L	1	-	-
Y7*	11	3	4	K95E	1	2	-	Q164P	1	1	-
Y7H	-	-	1	I96I	-	1	-	F165L	-	1	-
A8A	9	32	18	I96M	-	1	1	Y166*	1	2	2
A8I	2	-	-	I96N	-	1	-	Y166N	1	-	-
P9P	1	-	-	I96S	-	1	-	Y166L	1	-	-
H10N	3	1	-	D97A	-	1	-	M167I	1	-	1
H10P	1	-	3	D97E	1	4	2	M167R	8	2	2
H10Q	1	1	3	D97G	-	-	2	M167T	1	-	1
H10R	-	1	-	E98D	-	1	-	I169I	9	1	-
E11E	3	-	1	E98E	-	1	-	K170K	1	-	-
E11K	-	2	-	E98G	-	1	-	K170N	1	-	-
E11G	-	-	1	E98K	-	-	1	T171P	1	1	-
V12I	-	3	-	E98Q	-	-	1	T171T	1	1	-
V12E	-	-	1	A99P	-	1	1	N172I	-	1	-
V12G	-	1	-	L100I	-	1	-	N172K	24	3	1
V12Q	-	-	1	L100L	-	-	2	N172Q	1	-	-
I13F	2	2-	11	S101*	-	1	-	N173I	1	-	-
S14S	1	7	7	S101P	-	-	1	N173K	21	4	3
S14F	2	4	1	S101S	1	1	1	N173T	1	-	-
Q15H	8	7	3	S101T	-	-	3	K174N	-	1	-
Q15P	-	-	3	F102H	-	-	1	K174R	-	-	1
Q16K	-	2	-	F102L	-	3	-	Q175P	-	1	-
Q16Q	1	6	2	F102S	1	-	-	Q175L	1	-	-
Q16R	-	1	1	Y103*	-	1	1	Q175Q	1	-	-
E18*	-	2	-	Y103F	-	1	-	Y176*	3	-	-
E18G	-	1	-	Y103H	-	-	1	Y176K	1	-	-
E18K	3	15	8	Y103R	-	-	1	Y176N	1	-	-
R19H	-	1	-	Y103S	-	1	-	N177I	14	-	1

R19I	-	2	-	Y103Y	1	-	-	N177T	-	-	1
R19K	2	8	7	D104D	-	1	1	S178P	-	1	-
R19R	-	1	-	D104E	6	4	1	S178S	2	-	-
V21F	-	2	4	D104G	-	1	-	P179P	3	4	8
V21G	-	1	1	S105N	3	2	9	E181D	-	-	1
V21I	-	-	1	S105R	5	1	-	E181E	1	-	-
V21V	1-	25	9	S105S	1	-	-	M182R	1	-	-
D22E	-	1	1	K106*	-	1	-	K183E	2	-	-
D22G	1	-	-	K106K	1	-	-	K183R	-	-	1
K23Q	-	-	1	K106I	1	-	-	E184D	1	-	-
Y24N	-	1	-	K106R	-	-	1	E184E	-	2	1
V25F	1	7	5	K107K	-	1	-	E184G	2	-	-
V25G	-	1	-	K107M	4	1	6	R185K	2	1	-
V25V	-	3	1	N108K	7	1	6	Q187R	1	-	-
D26E	-	6	3	N108M	-	3	-	V188V	2	-	-
D26Y	-	-	1	N108N	-	-	1	F189L	1	-	-
R27F	-	1	-	N108R	1	-	-	L190*	1	-	-
R27I	-	2	-	N108S	6	1	1	L190F	1	-	-
R27K	4	1-	13	D109A	1	2	2	N192K	1	-	-
I29F	1	7	-	D109D	5	1	6	A193A	6	3	2
L30F	-	2	-	D109G	2	3	-	A193G	-	1	-
K31K	34	63	31	D109V	3	-	-	A193P	1	-	-
K31*	-	1	-	I110*	1	-	-	P194H	3	3	3
N32K	6	28	24	I110K	3	-	-	K195K	4	1	2
K33Y	-	1	-	I110L	3	6	3	K195Q	1	-	-
K33R	-	-	1	I110M	1	-	-	V196E	1	-	-
S35L	-	4	-	I110Q	1	-	-	V196G	-	-	1
S35N	-	1	-	N111D	1	-	-	N197Y	-	-	1
S35P	-	-	1	N111H	2	-	1	M198L	6	-	1
S35S	1	12	6	N111I	-	3	-	M198V	1	-	1
S35T	-	2	-	N111Y	7	1	1	H199H	1	-	-
L36L	-	-	-	K112*	1	3	-	H199L	14	2	5
L37*	2	7	5	K112A	-	-	1	H199Q	2	1	-
L37V	-	1	1	K112E	1	2	1	H199R	-	-	1
V38D	-	1	-	K112I	3	-	-	N200T	8	-	-
V38F	-	1	1	K112N	1	-	2	N201H	1	-	-
V38G	-	1	-	K112Q	1	-	-	N201I	3	1	-
V38Y	-	-	1	K112T	2	-	1	N201K	3	2	-
I39I	1	3	1	K112Y	1	-	1	N201S	1	-	-
I39N	-	-	1	Y113C	-	-	1	N202D	-	-	1
S41L	-	1	-	Y113F	-	1	-	N202K	5	1	-
S41P	1	-	-	Y113N	3	-	5	N202Y	-	-	1
S41S	1	4	1	N114D	3	1	3	K203K	-	1	-

L42F	-	-	1	N114E	-	1	-	N204K	2	1	-
L42L	-	1	-	N114F	1	-	-	S205G	-	1	-
S43F	-	-	1	N114G	1	1	-	S205I	1	-	-
S43P	-	1	-	N114H	11	8	5	Y207*	1	-	1
S43S	8	19	7	N114N	4	-	5	Y207F	7	-	-
S43T	-	-	1	N114P	2	-	-	K209K	2	1	-
V44G	-	1	1	N114R	1	-	-	E210D	2	2	-
V44V	1	2	1	N114T	5	3	1	E210G	5	-	-
L45P	-	-	1	N114Y	1	-	-	E210N	1	-	1
S46S	2	2	4	E115A	1	-	-	N212Y	1	-	-
V47E	-	2	1	E115D	1	-	-	R213G	1	-	-
V47F	-	-	1	E115E	2	-	-	R213K	1	-	-
V47I	-	-	2	E115G	2	1	1	R213R	1	-	-
V47V	27	78	28	G116G	5	2	3	T218S	1	-	-
V48C	-	1	-	G116R	-	-	1	Y219N	1	-	-
V48F	-	-	1	N117D	5	6	3	Y219Y	1	-	-
V48G	-	2	-	N117H	3	2	2	H220H	2	-	-
V48V	2	6	1	N117K	-	1	-	H220L	1	-	-
G49G	1	5	-	N117L	-	-	1	H220P	1	-	-
F50L	1	2	-	N117Y	4	2	-	H220Q	5	14	5
V51F	1	4	1	N118N	3	-	5	E221D	3	-	-
V51I	29	72	27	N118T	3	2	3	E221E	1	-	-
L52*	-	1	-	N119D	3	-	-	E221G	27	6	4
L52F	-	2	-	N119H	3	2	1	K223*	2	1	-
F53S	-	1	-	N119T	5	3	2	N224T	1	1	-
Y54F	-	2	-	N119Y	-	-	1	K225Q	-	1	-
Y54I	-	1	-	N120H	2	2	-	Y226I	1	-	-
Y54N	-	1	1	N120I	1	-	1	L227F	1	-	-
F55F	-	2	1	N120K	1	-	-	L227L	8	2	-
F55L	-	1	-	N120T	1	-	-	S228I	2	-	-
T56A	-	1	-	N120Y	2	1	5	S228N	2	-	-
T56F	-	1	-	N121G	1	1	-	S228S	1	-	-
T56P	1	-	-	N121H	4	-	5	S228T	1	1	1
T56S	-	2	1	N121I	2	-	-	S231T	-	-	-
T56T	5	13	9	N121K	1	2	-	S232P	1	1	-
P57P	-	5	6	N121M	-	2	-	S232S	1	-	-
N58L	1	-	-	N121R	-	-	1	K233N	1	-	-
N58H	-	-	1	N121S	1	-	1	P234P	5	3	1
N58S	-	-	2	N121Y	1	1	1	K236R	1	-	-
S59F	2	18	6	A122A	8	4	5	S238S	-	1	1
S59S	2	3	3	A122G	-	2	-	Y240F	2	-	-
R60K	14	29	18	D123A	-	-	1	L241F	2	2	-
R60I	-	-	1	D123D	3	-	5	L242*	2	-	-

K61N	-	1	-	F124L	1	-	-	D243D	-	-	1
S62C	-	1	-	K124*	-	1	-	Q244R	1	-	-
S62N	-	1	-	K124K	-	2	-	N246D	5	-	-
D63F	-	1	-	K124R	2	-	1	N246I	6	2	2
D63G	-	1	-	G125G	1	-	-	Y247C	1	-	-
D63V	1	9	2	G125V	1	-	-	Y247F	4	3	1
L64F	-	6	5	L127L	5	3	-	Y247L	-	-	1
K66*	-	1	2	L127P	-	1	-	E248E	-	1	-
S68F	-	-	1	L127Q	1	-	-	E248G	7	2	2
S68L	-	-	1	S128N	-	2	-	E249G	6	-	1
S68P	1	1	2	S128S	-	-	1	I251L	7	7	1
S69P	1	-	-	S128T	1	-	-	K252Q	1	-	-
S69S	-	1	3	F130T	-	1	-	K253N	2	2	2
V70F	1	-	1	K131*	-	2	-	Y254F	2	1	-
V70I	-	-	3	K131I	2	-	-	Y254L	-	-	1
V70V	3	-	-	K131K	1	1	-	Y254N	1	-	-
E71K	-	-	1	K131Q	1	-	-	K255G	-	-	1
N72I	1	-	-	K131R	-	-	1	K255K	1	1	2
N72K	1	4	3	E132G	-	1	-	K255R	8	7	5
N73A	1	-	-	N133H	1	-	1	G256G	1	1	2
N73I	1	-	-	T134A	-	-	1	N257D	3	4	-
N73K	1	2	2	T134K	1	-	1	N257E	3	2	4
N73N	1	-	-	T134P	1	4	2	N257K	2	1	1
N74I	1	2	-	T134T	3	4	3	E258G	-	1	1
N74K	1	6	5	P135P	4	2	-	E258K	2	2	-
N74R	-	-	1	S136P	1	1	-	F260F	9	4	12
N74S	-	2	-	S136S	-	2	-	D261	-	-	1
D75E	3	3	4	N137D	2	-	-	H262L	1	-	-
D75G	-	3	3	N137H	4	-	2	H262Q	1	-	-
D75V	-	2	-	N137K	1	-	1	A263A	1	1	-
D76A	-	1	-	N137Y	1	-	-	A264V	1	-	-
D76P	1	-	-	N138F	-	1	-	Y265Y	8	8	5
Y77C	-	-	1	N138I	6	-	-	D266D	8	8	6
Y77H	-	-	1	H141H	4	-	-	W267G	1	-	-
Y77N	2	3	3	H141L	1	-	-	R268K	1	-	1
Y77P	1	-	-	H141Q	5	3	-	L269*	1	1	2
I78*	1	-	-	N142K	13	3	1	L269I	1	-	-
I78I	-	-	1	K143K	-	1	-	H270H	8	6	3
I78L	3	2	1	K143R	1	-	-	H270P	-	1	2
I79I	-	1	-	K143T	1	-	-	H270Q	1	-	-
I79K	6	16	7	D144E	-	1	-	S271R	2	2	-
I79L	1	-	-	D144N	1	-	-	G272G	1	2	1
I79V	-	1	-	Y145H	1	-	-	V273E	1	-	-

N80C	-	-	1	Y145N	2	1	-	T274T	-	-	1
N80D	-	-	1	F146L	1	-	-	D278D	-	-	1
N80K	1	1	3	I147I	-	1	-	C282W	4	5	1
S81A	-	-	1	I147K	7	2	1	C285G	1	4	-
S81N	-	-	1	N148I	-	1	-	W286G	-	1	-
S81S	1	-	-	F150*	1	-	-	F288L	-	1	1
S81T	-	1	1	D151D	1	-	-	S289R	-	1	1
L82V	-	1	-	D151E	57	8	19	S290K	-	-	1
L82W	-	2	3	N152K	12	2	2	S290N	1	-	-
L83L	-	1	-	F154F	1	-	1	S290R	-	1	-
L83P	-	1	1	L155*	2	1	-	I291K	1	5	3
L83R	-	4	1	L155I	-	-	1	G292S	1	-	-
L83V	-	-	3	L155L	1	1	1	S293P	-	1	-
K84K	1	1	-	L155S	1	-	-	V294E	-	1	1
K84Q	1	-	-	L155W	1	-	-	E295K	1	2	2
S85C	-	-	1	M156I	1	-	-	S296P	-	-	1
S85T	-	-	1	M156K	2	-	1	S296S	1	2	2
P86L	-	1	-	M156R	3	-	-	R301G	-	-	-
P86P	1	2	5	M156V	2	-	-	R301K	-	-	-
N87K	6	9	4	N157H	1	-	-	R301R	1	1	1
N87S	-	2	-	N158K	8	1	2	N303I	-	1	-
G88G	1	1	-	N158S	2	1	3	L308F	-	1	-
K89K	1	1	1	A159A	-	-	1	E310G	-	1	-
K89R	-	1	1	A159G	1	2	1				

Supplementary Table 3 Novel and known *pfcoronin* mutations observed in all the Ghanaian isolates analyzed.

Mutation	Ecological zones/n			Mutation	Ecological zones/n		
	CS	FZ	GS		CS	FZ	GS
W45G	-	1	-	L90*	1	-	-
V47V	2	-	1	A91A	-	-	1
E48E	-	-	1	S92*	1	-	-
M52I	-	-	1	E95G	1	-	-
I53M	-	-	1	M97K	-	-	1
I56N	-	-	1	R100R	1	-	-
L58K	-	-	1	I101K	-	-	1
L58*	-	-	1	I101R	-	-	1
V62L	-	-	1	I104R	1	-	-
N64S	1	1	-	I104T	-	-	1
V67A	-	-	1	R105C	-	-	1
I68T	-	-	1	H106Q	-	-	1
L70M	-	-	1	D108E	-	-	2
H73Q	-	-	1	E109E	-	-	1
T74T	2	2	4	V111E	-	-	1
S75C	-	-	1	N112K	-	-	1
**P76S**	**6**	**2**	**14**	P117P	-	-	1
I77V	-	1	1	C119R	-	-	1
L78P	-	-	1	I120V	1	-	-
D79K	-	-	1	I120K	-	-	1
D79V	-	-	1	N122K	-	-	1
L80C	-	1	-	N122N	-	-	1
L80M	-	-	1	K126K	1	-	1
S81*	1	-	-	V128E	-	-	1
N83D	-	1	-	N129K	-	-	1
Y86D	-	1	-	L131L	-	-	1
Y86K	-	-	1	M136V	-	-	1
I89K	-	-	1	S143S	-	-	1

Supplementary Table 4 Novel and known *pfarps10* mutations observed in all samples analysed

	Ecological zones/n				Ecological zones/n		
**Mutation**	**CS**	**FZ**	**GS**	**Mutation**	**CS**	**FZ**	**GS**
H126W	1	-	-	S131X	1	-	-
H126R	1	-	-	S131E	1	-	-
H126M	-	2	3	S131V	1	1	-
H126C	-	1	-	S131*	1	-	1
H126L	-	-	2	S131N	-	2	2
V127W	1	3	2	R132*	1	-	-
**V127M**	**-**	**-**	**1**	R132S	1	-	-
V127D	1	-	-	R132A	1	1	-
V127G	-	1	1	R132K	1	1	1
V127C	-	1	1	E133R	1	-	-
V127*	-	-	1	E133N	1	-	-
D128I	1	1	2	E133S	1	-	1
D128K	1	-	1	Q134X	1	1	-
D128N	2	1	-	Q134A	1	-	-
D128G	-	1	-	Q134P	1	-	-
K129K	1	-	2	Q134H	1	-	-
K129T	1	-	-	Q134N	1	-	1
K129D	1	-	1	F135R	1	-	-
K129*	-	2	-	F135S	1	-	-
K129M	-	1	-	E136N	1	-	-
D130T	2	1	-	I137K	1	-	-
D130X	1	-	-	K138T	1	-	-
D130S	1	-	1	Q139V	1	-	-
D130N	-	-	2	Y140T	1	-	-

n: number of samples with the mutation, **bold**: known mutation

Supplementary Table 5: Novel and known *pffd* mutations observed in all isolates analysed.

Mutation	Ecological zones/n		
	CS	FZ	GS
A145S	-	-	1
K146*	-	-	1
V148G	1	-	2
E149K	-	-	1
V152V	1	-	-
D161Y	-	-	1
E162D	-	-	1
E163D	-	-	1
Q164H	-	-	1
T174T	-	-	1
P177P	-	-	1
V182V	-	-	1
K187N	-	-	1
D189H	-	-	1
M194V	-	-	1

Supplementary Table 6: Novel and known *pfmrp1* mutations observed in all isolates analysed.

Mutation	FZ	GS	CS	Mutation	FZ	GS	CS	Mutation	FZ	GS	CS
C861K	1	-	-	I884I	-	-	2	N866T	1	-	-
C894S	-	-	1	I888K	-	-	1	N866Y	14	4	13
D864G	17	4	1-	I899F	-	-	1	N867F	1	-	-
D864M	1	1	1	K886K	1	-	4	N891L	-	-	1
D864S	1	-	-	K887R	-	-	2	S897I	-	-	1
D864*	1	-	-	K895L	-	-	1	S885G	1	-	-
D865*	16	4	11	L862*	3	-	-	S885K	3	5	3
D865E	-	-	1	L862G	-	1	-	S885N	2	-	2
D865I	1	-	1	L862S	9	1	3	S885P	-	-	2
D865S	-	1	-	L862V	2	2	2	S885R	-	1	3
D865V	-	1	-	L872*	-	-	1	S885S	-	-	1
D874D	-	-	1	L88-F	-	-	1	S885T	32	7	14
D897E	-	-	1	L88-V	-	-	1	S89-C	1	-	-
D917R	-	1	-	L892L	-	-	1	S89-P	-	-	1
E896K	-	-	1	M863G	1	-	-	S9--P	-	-	1
F868I	1	-	-	M863R	1	1	-	T878T	-	-	1
F877L	-	-	1	M863W	4	1	2	T878Y	-	-	1
F901S	-	-	1	M863Y	18	1	9				
I876V	2	4	2	N866M	3	3	2				

Supplementary Table 7: Novel and known *pfmrp2* mutations observed in all isolates analysed.

Mutation	FZ	GS	CS	Mutation	FZ	GS	CS	Mutation	FZ	GS	CS
N513H	1	-	-	I568F	-	-	1	D629N	2	-	-
N514I	1	-	-	I568L	1	-	-	D630G	1	-	-
N514S	-	1	-	L572F	1	-	-	D631G	5	-	3
N517M	1	-	-	I573K	-	-	1	D633N	3	-	-
N518N	1	-	-	E574K	2	-	-	D635V	1	-	-
I519I	1	-	-	N577D	-	-	1	Y636C	2	-	-
S520S	1	-	-	K580T	1	-	-	D637N	2	-	-
I523M	1	-	-	K580S	1	-	-	N641D	1	-	-
V524G	1	-	-	N583I	1	-	-	N642T	-	-	1
F529S	1	-	-	K584R	-	-	1	N642S	1	-	-
F530S	1	-	-	K584I	-	-	1	N645D	6	-	4
Y532S	1	-	-	N587H	-	-	1	D647D	1	-	-
I533L	3	-	-	Y588*	-	-	1	Y648F	-	-	1
R534K	2	-	-	Y589*	-	-	1	D649N	1	-	1
K538K	1	-	-	Y589I	1	-	-	M652V	1	-	1
N540I	1	-	-	N593S	1	-	-	M657T	1	-	-
N540S	1	-	-	Y598F	1	-	-	K672R	-	1	-
S543G	1	-	-	K600K	1	-	1	K681E	1	-	-
S544T	2	-	1	L602V	-	1	-	N686H	1	-	-
S545R	1	-	-	N604S	1	-	-	N686Y	1	-	-
S545C	1	-	-	M605V	-	-	1	Q694H	2	-	-
S547S	-	1	-	K606E	-	-	1	L699L	1	-	-
L548F	1	-	-	S607G	1	-	-	H703L	1	-	-
I549T	1	-	-	N609K	1	-	-	K704H	1	-	-
T550P	2	-	-	T610T	1	-	-	T706A	1	-	-
T550T	1	-	-	C611C	-	-	1	K714I	5	-	2
Y555Y	1	-	-	C611G	1	-	-	K715Q	1	-	-
K556N	1	-	-	F613V	-	-	1	N719D	1	-	-
K556T	1	-	-	Q614L	1	-	-	K723E	1	-	-
L558F	1	-	-	N615K	-	-	1	K743I	1	-	-
I559S	1	-	-	N617P	-	-	1	K776R	1	-	-
V562G	1	-	-	N617D	1	-	-	N783H	1	-	-
V562V	1	-	-	N619T	-	-	1	G794R	1	-	-
V562A	1	-	-	N619P	-	-	1	F801I	1	-	-
N563D	-	-	1	N620S	1	-	-	Y802N	1	-	-
N563H	1	-	-	N622S	2	-	-	L805L	1	-	-
N563Y	-	1	1	G623D	1	-	-	G806R	1	-	-
N564Y	1	-	-	Y624C	1	-	-	L811*	1	-	-
P566P	-	-	3	D625Y	-	-	1				
N567H	-	-	1	D627G	-	1	1				

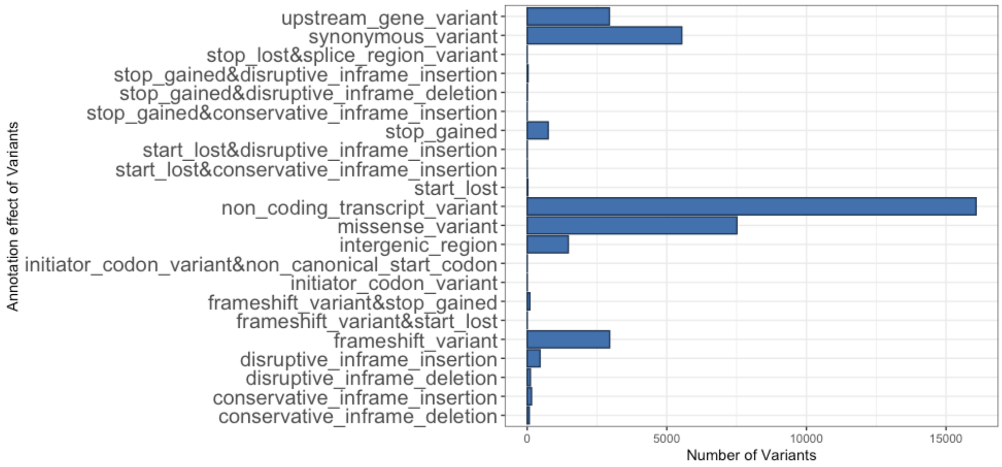

Supplementary Figure 1: Distribution of annotation effects of all genetic variants observed in *pffp2* in Ghanaian isolates.
